# A new characteristic of SLE: subclinical vein involvement–an in-depth biochemical and imaging study

**DOI:** 10.1093/rheumatology/keaf468

**Published:** 2025-09-05

**Authors:** Derya Yildirim, Abdulsamet Erden, Nemat Ibrahimkhanli, Elena Elefante, Rahime Duran, Handenur Koc Kanik, Rıza Can Kardas, İbrahim Vasi, Hazan Karadeniz, Mahinur Cerit, Halit Nahit Sendur, Marta Mosca, Hamit Kucuk, Mehmet Akif Ozturk, Abdurrahman Tufan

**Affiliations:** Sincan Education and Research Hospital, Rheumatology Clinic, Ankara, Turkey; Department of Rheumatology, Gazi University Faculty of Medicine, Ankara, Turkey; Department of Rheumatology, Gazi University Faculty of Medicine, Ankara, Turkey; Department of Radiology, Ultrasonography Unit, Gazi University Faculty of Medicine, Ankara, Turkey; Department of Clinical and Experimental Medicine, University of Pisa, Rheumatology Unit, Pisa, Italy; Department of Rheumatology, Gazi University Faculty of Medicine, Ankara, Turkey; Faculty of Medicine, Department of Rheumatology, Ankara University, Ankara, Turkey; Department of Rheumatology, Gazi University Faculty of Medicine, Ankara, Turkey; Department of Rheumatology, Gazi University Faculty of Medicine, Ankara, Turkey; Rheumatology Clinic, Gulhane Education and Research Hospital, Ankara, Turkey; Department of Radiology, Ultrasonography Unit, Gazi University Faculty of Medicine, Ankara, Turkey; Department of Radiology, Ultrasonography Unit, Gazi University Faculty of Medicine, Ankara, Turkey; Department of Clinical and Experimental Medicine, University of Pisa, Rheumatology Unit, Pisa, Italy; Department of Rheumatology, Gazi University Faculty of Medicine, Ankara, Turkey; Department of Rheumatology, Gazi University Faculty of Medicine, Ankara, Turkey; Department of Rheumatology, Gazi University Faculty of Medicine, Ankara, Turkey

**Keywords:** SLE, vein wall thickness, thrombosis

## Abstract

**Objectives:**

SLE is a heterogeneous autoimmune disorder often complicated by vascular events, with or without antiphospholipid antibody syndrome (APS). This study aimed to explore subclinical venous involvement in SLE using biochemical and imaging modalities, focusing on vein wall thickness (VWT) and inflammation-related biomarkers.

**Methods:**

In this cross-sectional study, 68 SLE patients were categorized based on antiphospholipid antibody (APA) status and clinical APS. Results were compared with 22 RA patients and 20 healthy controls. Serum levels of P-selectin, growth differentiation factor 15 (GDF15) and citrullinated histone 3 (CH3) were measured using ELISA. Ultrasonographic assessments evaluated VWT at bilateral jugular veins, femoral veins, portal vein and femoral artery. Correlations and predictors of vascular changes were analysed statistically.

**Results:**

VWT was significantly increased in SLE patients compared with both the control groups (*P* < 0.001), regardless of APA and APS status. Serum P-selectin, GDF15 and CH3 levels were elevated in SLE-APS patients. GDF15 levels correlated positively with VWT, and increased portal VWT was independently associated with thrombosis. Biomarkers showed significant associations with APS, suggesting their role as indicators of prothrombotic states. No significant associations were found between vascular parameters and disease activity score.

**Conclusion:**

Subclinical venous involvement appears to be a novel vascular feature of SLE, reflected by increased VWT and its association with thrombosis and biomarkers. Increased portal vein thickness may indicate vascular risk. Whether these changes result from inflammation or vascular remodelling remains unclear, but their presence irrespective of disease activity highlights their clinical relevance.

## Graphical Abstract

**Figure:**
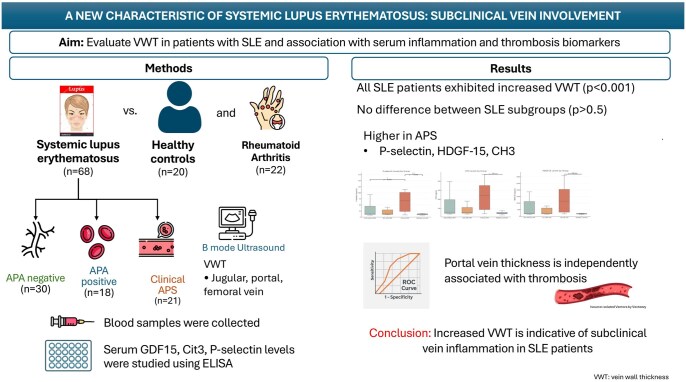

Rheumatology key messages

- Subclinical venous involvement in SLE, marked by increased vein wall thickness, occurs independently of disease activity.
- Portal vein wall thickness is independently associated with thrombosis, suggesting its potential as a vascular risk marker.
- Elevated P-selectin, GDF15 and CH3 correlate with venous changes, highlighting biochemical markers of thrombo-inflammatory processes.

## Introduction

Systemic lupus erythematosus (SLE) is a relapsing–remitting autoimmune disorder characterized by antibodies to nuclear and cytoplasmic antigens, multisystem inflammation and diverse clinical manifestations. Vascular events are one of the leading causes of death in SLE. Studies have shown that ∼23% of SLE patients may experience a thrombotic event during their lifetime [1]. Secondary APS is a well-known feature of SLE which affects 30–40% of patients. However, it does not alone explain the increased thrombosis, since 40% of patients with thromboembolic disease are negative for APA [1]. Therefore, hypotheses, such as seronegative APS, have been suggested [2]. Moreover, among the 30–40% of patients with SLE who produce APA, only 10% experience a thrombotic event [1].

In addition to thrombotic disease, vasculopathy resulting from endothelial dysfunction, cellular metabolic perturbations and the activation of inflammatory and thrombotic pathways may contribute to the vascular complications of SLE. Therefore, vascular inflammation markers have been utilized in numerous studies on SLE and are considered significant in its pathogenesis. Human Growth Differentiation Factor 15 (GDF-15), a member of a TGF-β superfamily, is a marker of oxidative stress, inflammation and tissue remodelling [3]. Recently, serum levels of GDF15 were proven as a close relationship with an increased risk of venous thromboembolism [4]. P-selectin is crucial for the initial adhesion of platelets and leukocytes during injury and inflammation, thus playing a significant role in hemostasis and thrombosis [5, 6].

Neutrophil extracellular traps (NETs) are formed by nuclear components and are one of the leading pathologies in the pathogenesis of SLE. NETs contribute to the pathogenesis of deep vein thrombosis by providing a stage for blood cells to adhere to and activate coagulation pathways [7]. It is believed that the plasma level of citrullinated histone H3 (CitH3), a specific marker of NETs, could help diagnose and predict DVT in cases of traumatic lower extremity fractures and lumbar fractures [8, 9]. In the pathogenesis of SLE vascular events, characterized by the thrombo-inflammation complex, it may also play a role as a marker of NETosis.

Beyond these biochemical markers, subclinical vein inflammation manifests as morphologic alterations in veins, such as increased vein wall thickness (VWT). Thickened vein walls are a feature of inflammatory diseases with thrombotic tendencies, such as Behcet’s disease and VEXAS syndrome [10, 11]. Increased VWT is independent of age, sex and received treatments, suggesting a primary role of inflammation [11]. However, VWT is not increased in all inflammatory diseases, indicating that only certain inflammatory conditions exhibit this relationship [11]. Considering the high incidence of venous vascular involvement in SLE, even in the absence of overt thrombotic events, we hypothesize that subclinical vein involvement may be a characteristic feature of the disease. Accordingly, this study aims to explore subclinical vein involvement in SLE using both biochemical and imaging modalities (ELISA and ultrasonographic evaluation).

## Materials and methods

### Study design and patient population

This was a prospective cross-sectional study in which patients were consecutively enrolled during their routine outpatient visits, and all data were collected prospectively at the time of evaluation. Among the SLE patients, 20 had APS secondary to SLE, 18 were APA-positive without APS and 30 were negative for both APA and APS. RA patients (*n* = 22) and healthy individuals (*n* = 20) were included as the disease and healthy control groups, respectively. The 2019 American College of Rheumatology/European League Against Rheumatism (ACR/EULAR) criteria for the diagnosis of SLE [12], the Sapporo criteria set for the diagnosis of APS [13] and the 2010 ACR/EULAR criteria set for the diagnosis of RA [14] were used. Patients over the age of 65, those with chronic conditions such as chronic kidney failure were excluded from the study. Also, patients with known cardiovascular risk factors such as hypertension, diabetes mellitus or hyperlipidaemia were excluded to avoid confounding influences on vascular measurements and biomarker levels. The institutional ethics committee approved the study (Gazi University Ethical Committee, 01–07-2022, number: 10192), and informed consent was obtained from all participants. All participants provided written informed consent prior to inclusion in the study, in accordance with institutional and national ethical standards.

Patients participated in this study by providing blood samples and relevant clinical data. However, they were not involved in the design, conduct, analysis or dissemination of the research. The authors declare no competing interests. This research did not receive any specific grant from funding agencies in the public, commercial or not-for-profit sectors. The article does not contain any AI-generated images or manipulated figures.

### Assessment of clinical activity

All patients diagnosed with SLE were evaluated for their demographic characteristics, organ involvement and clinical activities. Complete blood count, serum biochemistry, anti-dsDNA, complement 3 and 4 (C3, C4), IgG, APA levels, spot urine protein creatinine excretion, acute phase reactants and ESR were determined. The clinical activity in SLE was assessed using the SELENA-SLEDAI activity index. In the initial assessment, scores of 6 and above were considered clinically active, while score changes of 4 and above were deemed significant [15]. Patients with SLE but without APS who had documented vascular involvement were excluded from the study to avoid potential bias.

### Evaluation of biomarkers in serum

Venous blood samples were taken from SLE patients and healthy controls after overnight fasting. All blood samples and ultrasonographic assessments were performed on the same day as the clinical visit during which SELENA-SLEDAI scores were recorded, ensuring temporal alignment of laboratory, imaging and disease activity data. Blood samples collected for analysis were centrifuged at 4000 rpm in 10 min. Serum levels of P-selectin, GDF15 and citH3 were evaluated using ELISA (Bioassay Technology Lab, E7533Hu, Shanghai, China) according to the manufacturer’s instructions.

### Evaluation of ultrasonographic study of vessel thicknesses

Neck, liver and bilateral lower extremity venous Doppler ultrasonography was performed by an experienced radiologist blinded to the clinical data of all patients. Ultrasound examinations were performed on the same device (RS85 Prestige, Samsung, Medison Co. Ltd) and included the assessment of bilateral common and superficial femoral veins, internal jugular veins and portal veins. Patients were positioned in the supine position. Portal VWT was measured from the posterior wall of the main portal vein with a high-resolution curved array (1–7 MHz) transducer via intercostal approach. Common femoral, superficial femoral and internal jugular veins were examined with a high-resolution linear (2–14 MHz) transducer. The presence or absence of chronic thrombotic changes, recanalization and collateral formation in veins was evaluated. The common femoral VWT was measured at 2 cm distal from the saphenofemoral junction. Superficial femoral VWT was measured at 2 cm caudal from the femoral bifurcation. Internal jugular VWT measurements were obtained at the level of the cricoid cartilage. All measurements were done from the posterior wall of veins. Internal jugular vein measurements were performed both at the end of inspiration and expiration and mean values were recorded. Measurements were repeated twice by the same radiologist to assess intraobserver reliability.

### Statistical analysis

Statistical analyses were performed using SPSS version 21.0 (Chicago, IL, USA). Continuous variables were expressed as mean ± standard deviation (S.D.) or median and interquartile range (IQR), depending on data distribution. The normality of distributions was assessed using the Kolmogorov–Smirnov test. Serum levels of P-selectin, GDF15 and CitH3 were non-normally distributed, and comparisons between the groups were performed using the Kruskal–Wallis test, followed by Dunn’s test for *post hoc* subgroup analysis. Correlation analyses were conducted using Pearson’s or Spearman’s tests, as appropriate. A *P*-value of <0.05 was considered statistically significant.

Logistic regression analysis was employed to examine the association between vascular parameters and the presence of thrombosis. Variables showing statistically significant associations in multivariable models were subsequently re-evaluated using simplified univariable logistic regression. The discriminatory performance of these models was assessed via receiver operating characteristic (ROC) curve analysis, and the area under the curve (AUC) was reported. A type I error rate of 5% was used to determine the statistical significance of predictive performance.

In addition, multiple linear regression analysis was used to identify independent predictors of serum P-selectin, GDF15 and CitH3 levels. The adequacy of model fit was assessed using residual diagnostics and goodness-of-fit statistics. Sample size adequacy was determined by power analysis using R software.

### Patient and public involvement

Patients and the public were not involved in the design, conduct, reporting or dissemination plans of this research.

## Results

### Baseline characteristics

The study population comprised 68 patients with SLE, grouped based on APA status and clinical APS. Patients were categorized as follows: APS secondary to SLE (*n* = 20), APA-positive without thrombotic or obstetric complications (*n* = 18) and APA-negative (*n* = 30). In the clinical APS group, a total of nine patients had obstetric complications and 14 had thrombotic events. Three patients experienced both thrombosis and obstetric manifestations. Among the patients with thrombosis, arterial thrombosis was observed in nine individuals and venous thrombosis in five. Notably, five of the patients with arterial thrombosis had experienced cerebrovascular occlusion. Results were compared with healthy controls (*n* = 20) and patients with RA (*n* = 22). Comparison of demographic and laboratory parameters are present in Table 1. All patients included in the study were similar in terms of age and sex.

**Table 1.** Comparison of clinical and laboratory parameters between study groups

	SLE-APS (*n* = 20)	SLE w/(+) APA (*n* = 18)	SLE w/o APA & APS positivity (*n* = 30)	RA (*n* = 22)	Healthy (*n* = 20)	*P*
Age (years)^a^	41 (20)	43 (21)	38.5 (12)	42 (12)	30 (6)	0.6
BMI (kg/m^2^)^a^	25 (6)	24 (4)	25.5 (6)	26 (3)	24 (2)	0.09
Sex (female ratio, %)	100	94.4	93.3	92	100	0.1
Anti-dsDNA^a^	130 (32)	126 (36)	123.5 (36)	NA	NA	0.9
C3^a^	76 (18)	65 (25)	82 (23)	NA	NA	0.4
C4^a^	12 (9)	11 (10)	18 (7)	NA	NA	0.2
SLEDAI^a^	7 (6)	5 (4)	7 (7)	NA	NA	0.8
CRP(mg/l)^a^	5 (3)	5.2 (3)	4.4 (2)	5 (1)	3 (1)	0.8
ESR(mm/h)^a^	25 (6)	30 (21)	22 (16)	30 (11)	12 (8)	0.1
Haemoglobin(g/dl)^a^	12 (1.6)	12.8 (1.4)	13.5 (2.1)	13 (2.2)	13 (1.1)	0.051
WBC (number/Ul)^a^	5200 (2300)	6200 (4400)	6400 (4200)	5200 (1200)	5000 (2500)	0.052
Neutrophil (cell/ml × 10^4^)^a^	3300 (1900)	3950 (1500)	4000 (1900)	3000 (1200)	3200 (1200)	0.12
PLT(cell/ml × 10^4^)^a^	145 (65)	291 (46)	221.5 (12.5)	254 (24)	256 (80)	**0.02** ^b^
D-dimer (µg/ml)^a^	0.6 (0.4)	0.6 (0.3)	0.4 (0.3)	0.4 (0.1)	0.3 (0.1)	0.2
SDI^c^	2 (0–11)	0 (0–2)	0 (0–7)	NA	NA	**0.007** ^b^
P-selectin (ng/ml)^c^	73.9 (9.2–114.3)	12.4 (8.8–92.7)	14.4 (9.6–62.9)	NA	10.9 (4–49)	**<0.001** ^b^
GDF-15 (ng/ml)^c^	1323 (182–3109)	220 (165–1420)	211.3 (167.5–1550)	NA	186 (120–485)	**<0.001** ^b^
CH3 (ng/ml)^c^	295 (41–474)	51 (38–180)	53.4 (38–269)	NA	59 (28–164)	**<0.001** ^b^

a These numerics are presented as (median (IQR)).

b Difference is caused by patients with clinical APS group, whereas other groups had insignificant differences.

c These numerics are presented as (median (min–max)).

SLE w/(+) APA: SLE patients with antiphospholipid antibody positivity, SLE w/o APA & APS positivity: SLE patients without antiphospholipid antibody positivity and antiphospholipid syndrome, C: complement, SLEDAI: SLE disease activity score, WBC: white blood cells, PLT: platelet, SDI: SLE damage index, GDF-15: Human Growth Differentiation Factor 15, CH3: citrullinated histone 3, NA: not applied.

Classical cardiovascular risk factors such as hypertension, diabetes mellitus or hyperlipidaemia were not present in the study population. Eleven patients were using angiotensin-converting enzyme inhibitors (ACEi), prescribed specifically for the management of proteinuria rather than for blood pressure control. Regarding smoking status, current smoking was identified in two patients within the APA-positive non-APS group, two within the APA-negative group and one patient with clinical APS.

Neurological involvement was significantly higher in the clinical APS group (*P* = 0.008), driven by increased incidences of optic neuritis, epilepsy and cerebrovascular events. Cardiac involvement was limited to one case of cardiomyopathy, observed exclusively in the APS group. No significant differences in systemic or organ-specific involvement were noted across the SLE subgroups (Supplementary Table S1). Disease activity, as determined by SELENA-SLEDAI scores, was comparable between the groups and primarily driven by skin, joint and serological abnormalities.

### Vein wall thickness

VWT was significantly increased in SLE patients compared with both the healthy controls and RA patients, irrespective of APA or APS status (*P* < 0.001 for all; Table 2). Although APS patients demonstrated a trend towards greater VWT compared with APA-negative SLE patients, the difference did not reach statistical significance. No significant differences in VWT were observed when patients were stratified by 4 point change in SELENA-SLEDAI score (Supplementary Table S2). In addition to subgroup comparisons, a direct *post hoc* analysis was performed between the full SLE cohort (*n* = 68) and the control group (RA + healthy controls, *n* = 42). SLE patients exhibited significantly greater VWT across all venous regions (right/left CFV, SFV, JV and portal vein) compared with the controls (all *P*-values <0.01). This finding supports the generalizability of subclinical venous involvement as a characteristic of SLE independent of subgroup stratification (see Supplementary Table S3). To rule out treatment-related confounding, vascular measurements and serum biomarker levels were also compared between patients receiving or not receiving immunosuppressive agents and hydroxychloroquine at the time of sample collection. No significant differences were observed across treatment subgroups (*P* > 0.5, Supplementary Table S4).

**Table 2.** Comparison of vessel wall thicknesses between the study groups

	SLE-APS (*n* = 20)	SLE w/(+) APA (*n* = 18)	SLE w/o APA & APS positivity (*n* = 30)	RA (*n* = 22)	Controls (*n* = 20)	*P*
Common femoral vein (mm, median (min–max))						
Right	0.4 (0.3–1.3)	0.4 (0.3–0.7)	0.4 (0.1–0.7)	0.3 (0.2–0.4)	0.3 (0.2–0.4)	**<0.001** ^a^
Left	0.4 (0.3–1.1)	0.4 (0.3–0.6)	0.4 (0.1–0.6)	0.3 (0.2–0.5)	0.3 (0.2–0.5)	**<0.001** ^a^
Superficial femoral vein (mm, median (min–max))						
Right	0.4 (0.3–1.1)	0.3 (0.2–0.5)	0.4 (0.1–0.6)	0.2 (0.2–0.4)	0.3 (0.2–0.4)	**<0.001** ^a^
Left	0.4 (0.2–1.0)	0.3 (0.2–0.5)	0.4 (0.1–0.6)	0.2 (0.1–0.4)	0.3 (0.2–0.5)	**<0.001** ^a^
Portal vein (mm, median (min–max))	1.35 (1.1–3.1)	1.2 (0.9–1.5)	1.2 (1–1.7)	0.9 (0.2)	1 (0.8–1.6)	**<0.001** ^a^
Juguler vein (mm, median (min–max))						
Right	0.3 (0.2–0.8)	0.3 (0.2–0.4)	0.3 (0.2–0.5)	0.2 (0.2–0.3)	0.2 (0.2–0.5)	**<0.001** ^a^
Left	0.3 (0.2–0.7)	0.3 (0.2–0.4)	0.3 (0.2–0.4)	0.2 (0.2–0.2)	0.2 (0.2–0.5)	**<0.001** ^a^
Femoral artery (mm, median (min–max))						
Right	0.6 (0.4–0.9)	0.5 (0.4–1.1)	0.6 (0.5–0.8)	0.6 (0.4–0.7)	0.4 (0.4–0.6)	**<0.001** ^a^
Left	0.6 (0.5–0.8)	0.6 (0.4–1)	0.6 (0.5–0.8)	0.5 (0.5–0.7)	0.4 (0.4–0.8)	**<0.001** ^a^

a VWT in patients with RA and control groups was observed at similar levels and was lower compared with the SLE groups.

APS: antiphospholipid syndrome, APA: antiphospholipid antibody, min–max: interquartile range.

The SLE damage index (SDI) was significantly higher in the SLE-APS group (median: 2, range 0–11) compared with the other SLE subgroups (*P* = 0.007). Correlation analysis revealed that SDI positively correlated with several ultrasonographic vein wall measurements, most notably left jugular vein thickness (*r* = 0.47) and portal vein diameter (*r* = 0.38).

All assessed VWT measurements were positively correlated (Supplementary Table S5). Notably, the thicknesses of the bilateral common and superficial femoral veins, as well as femoral artery thicknesses, were positively associated with age (Supplementary Fig. S1 [Section S1]).

Among the vascular wall thickness parameters, several significant correlations were observed. GDF-15 levels were positively correlated with femoral artery wall thickness (*r *= 0.22, *P* = 0.04) and femoral VWT (*r *= 0.20, *P* = 0.04), while P-selectin showed a significant correlation with femoral VWT (*r *= 0.25, *P* = 0.02) (Supplementary Table S6). Notably, portal VWT was the only parameter that demonstrated a statistically significant positive correlation with all three biomarkers: GDF-15 (*r *= 0.27, *P* = 0.01), P-selectin (*r *= 0.26, *P* = 0.02) and CH-3 (*r *= 0.23, *P* = 0.04).

**Figure 1.**
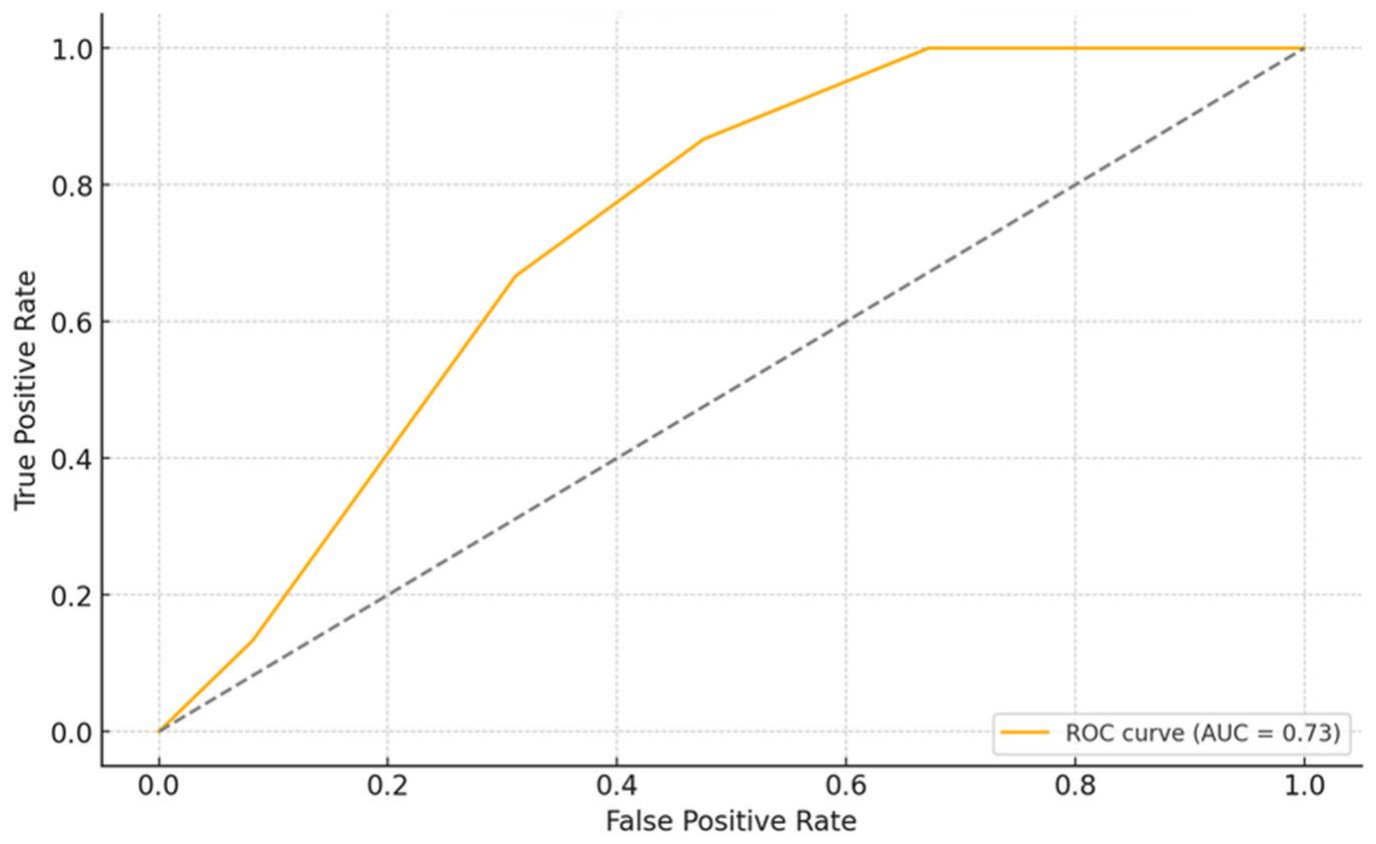
ROC curve demonstrating the discriminative ability of portal vein wall thickness for history of thrombosis

Multivariate logistic regression analysis was performed to evaluate the association of biomarkers (P-selectin, GDF-15, CH-3) and vascular wall thickness measurements (portal vein, mean femoral vein thickness and mean femoral artery thickness) with the presence of thrombosis and antiphospholipid antibody (aPL) positivity. Among these parameters, portal VWT showed a statistically significant association with the presence of thrombosis (*P* = 0.001). No significant associations were observed between other variables and thrombosis or aPL positivity. In light of this finding, a univariate logistic regression analysis was subsequently conducted to further explore the relationship between portal vein thickness and thrombosis. This association remained significant when re-tested in a simplified univariable model (*P* = 0.007). The univariable model demonstrated fair discriminative ability with an AUC of 0.70 based on ROC curve analysis (OR = 2.01, 95% CI: 1.21–3.36, Table 3, Fig. 1).

**Table 3.** Multivariate and univariate regression analyses of vessel wall thickness and biomarkers (dependent variable is thrombosis)

Variable	Coef.	Std.Er	*z*	*P*	OR	Confidence interval
const	−5.89	2.60	−2.18	0.02	0.002	0–0.54
Mean SFV^a^	2.69	4.30	0.62	0.53	14.805	0.003–7.4
Mean FA^b^	−3.32	4.50	−0.74	0.46	0.036	1.0–25.1
Portalvein	3.83	1.1	3.48	0.0005	46.2	5.6–382.0
p_selectin	0.034	0.023	1.46	0.14	1.0346	0.98–1.08
HGDF15	0.003	0.002	1.19	0.23	1.003	0.99–1.0
CH3	−0.019	0.014	−1.33	0.18	0.9812	0.95–1.0

a Mean SFV refers to the average of right and left superficial femoral vein wall thickness.

b Mean FA refers to the average of right and left femoral artery wall thickness.

HGDF: human growth differentiation factor, CH3: citrillunated histone 3, OR: odds ratio (exp(coefficient)), Coef.: regression coefficient (log(OR)), 95% confidence interval for the OR.

### Serum biomarkers

**Figure 2.**
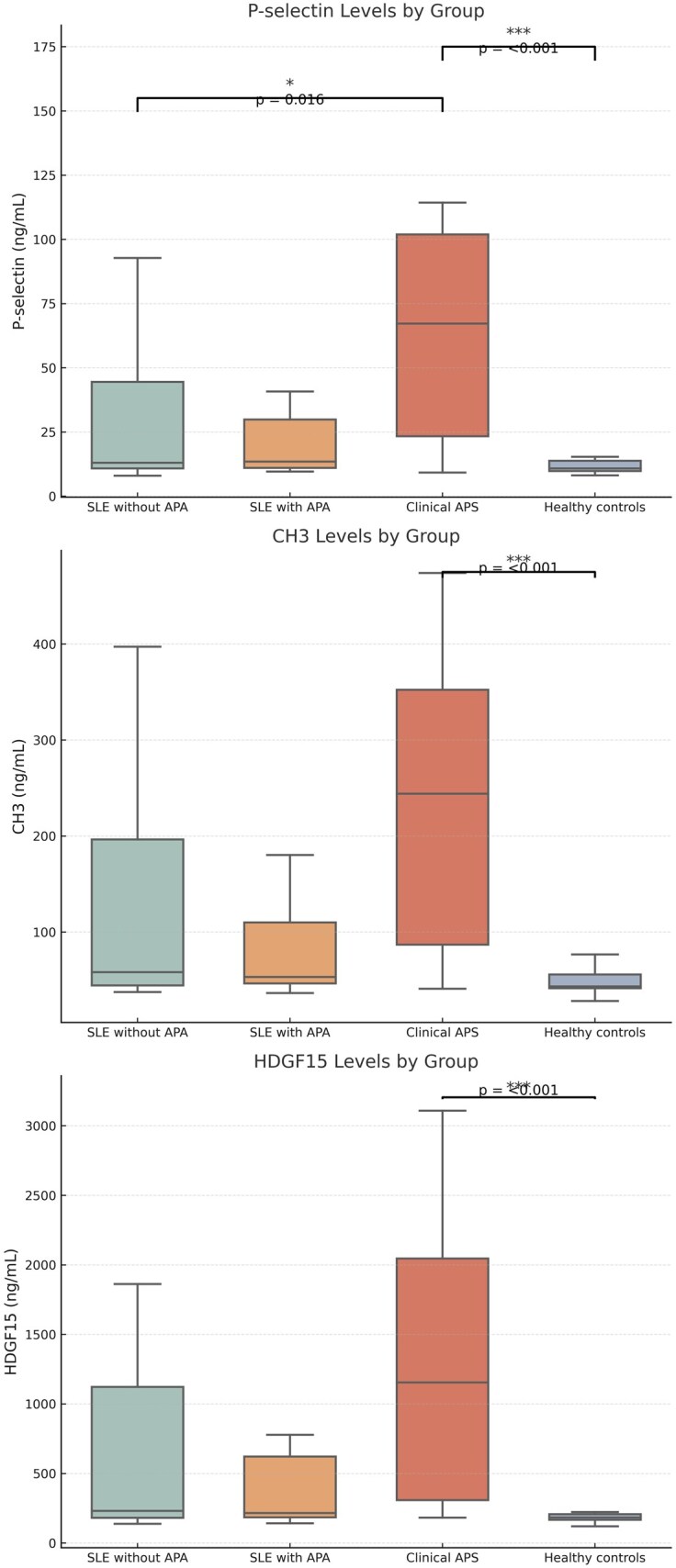
Comparison of serum P-selectin, GDF15 and CH3 levels among SLE subgroups and healthy controls

Serum levels of P-selectin, GDF15 and CH3 were significantly elevated in the APS group compared with both the non-APS SLE patients and the controls (*P* < 0.001 for all; Fig. 2). No significant differences in these biomarkers were noted between the healthy controls and the SLE patients without APS. D-dimer levels did not differ significantly across the groups. Biomarker levels were unaffected by clinical activity evaluated with SELENA-SLEDAI scores (Supplementary Table S2). Also, no correlation was found between SDI and circulating biomarkers including P-selectin, GDF15 and CH3.

Significant positive correlations were observed among CH3, GDF15 and P-selectin levels. GDF15 and CH3 levels were also positively correlated with BMI (Supplementary Fig. S1 [Section S2]). Among APA subtypes, GDF15 and P-selectin levels were significantly associated with anti-beta-2 glycoprotein I (IgG) and anticardiolipin antibodies (ACA; IgG and IgM), while CH3 levels showed an additional correlation with anti-beta-2 glycoprotein I (IgM) (Supplementary Table S6).

**Figure 3.**
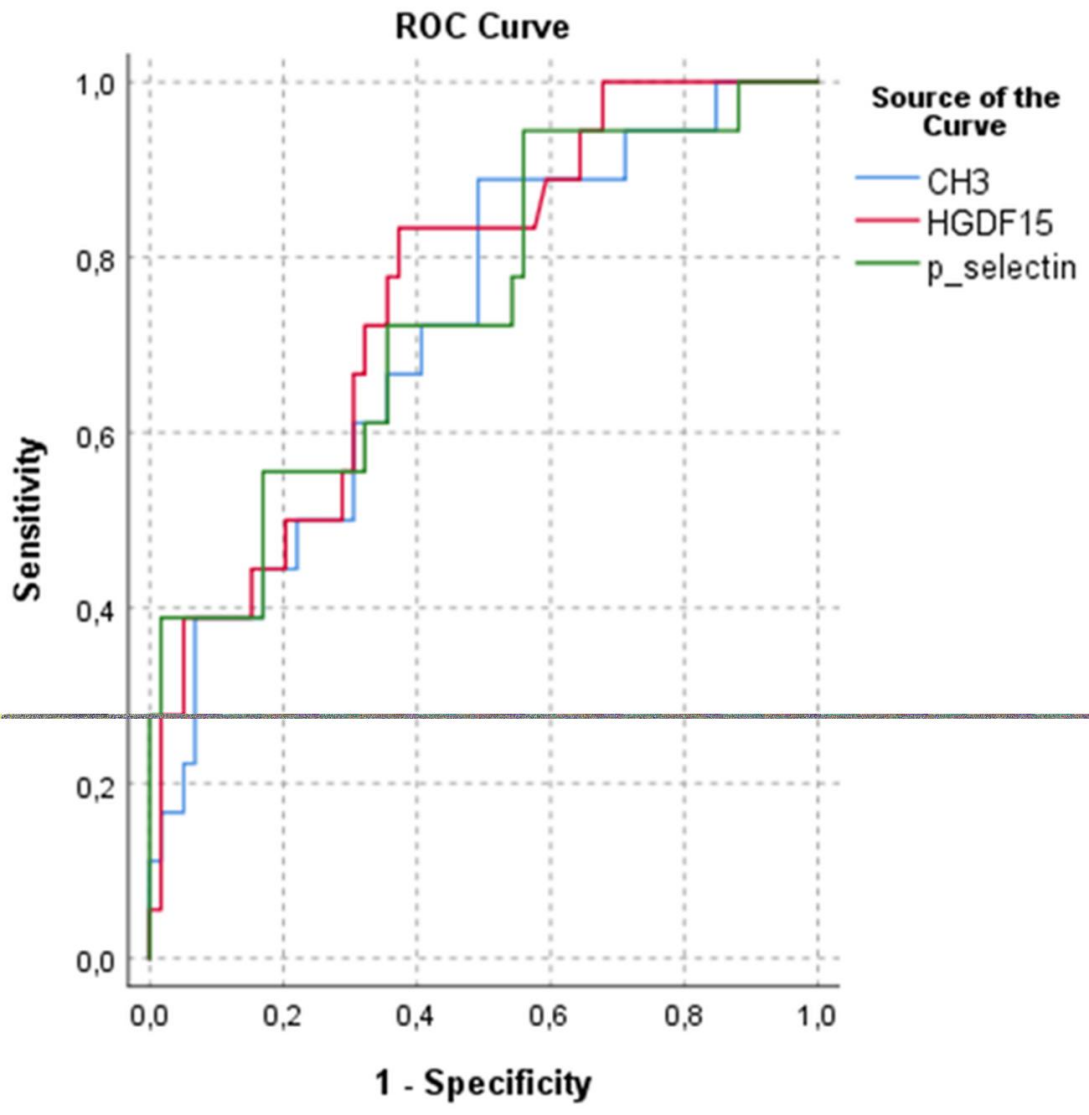
ROC curves of P-selectin, GDF15 and CH3 for predicting increased vein wall thickness in the APS subgroup

In the APS subgroup, ROC analysis revealed that P-selectin (AUC: 0.74, 95% CI: 0.61–0.88), GDF15 (AUC: 0.76, 95% CI: 0.64–0.88) and CH3 (AUC: 0.72, 95% CI: 0.59–0.85) demonstrated fair discriminative ability in detecting increased VWT. All three biomarkers showed a sensitivity of 66%, with specificity ranging from 64% to 67% (Fig. 3).

## Discussion

This study highlights a novel aspect by demonstrating that vessel wall thickening is a marker of subclinical vein inflammation in SLE patients, regardless of APA or APS status. VWT was notably increased in the SLE patients compared with the healthy controls and RA patients, emphasizing the unique vascular involvement in SLE. Additionally, elevated levels of biomarkers such as P-selectin, GDF15 and CH3 were observed in SLE-APS patients, suggesting their potential as indicators of risk for thrombotic disease. These findings provide critical insights into the venous involvement in SLE and underscore the potential of integrating biochemical and imaging modalities for early detection and monitoring of vascular complications in this population.

The significant positive correlations observed between serum GDF15 levels and VWT across multiple venous sites reinforce the hypothesis that systemic inflammation plays a critical role in vascular remodelling in SLE. HGDF-15, a stress-inducible protein belonging to the TGF-β cytokine superfamily, has an increasingly data about its involvement in inflammation and angiogenesis [16]. Emerging research highlights the connection between GDF-15 and cardiovascular events, subclinical atherosclerosis and its potential as a predictor of coronary artery disease and cardiovascular mortality in the general population [17, 18]. In a recent study, they showed a relationship between carotis intima media thickness and APS [17]. The positive correlation with VWT may indicate the potential role of GDF-15 in thrombo-inflammatory mechanisms involving aPL-mediated endothelial cell activation. The association between GDF15 and APA positivity may support its role as a marker of prothrombotic states in SLE, particularly in the context of APA-mediated vascular damage. Comparable findings of venous wall thickening have been described in Behçet disease [19], supporting the notion that venous structural changes may represent a broader feature of systemic vasculitides.

In this context, it is worth noting that LA testing in our study was analysed as a continuous variable, based on the raw numeric output of phospholipid-sensitive coagulation assays. Although this approach is not conventional in clinical practice, it allowed for the evaluation of potential dose-dependent effects or subclinical thresholds of procoagulant activity that may not be captured using binary classification. We acknowledge that further studies are warranted to assess the validity and reproducibility of this strategy in larger cohorts. Taken together, these results raise the possibility that vascular inflammation in SLE represents a distinct pathological process, potentially requiring targeted therapeutic strategies.

Furthermore, P-selectin and CH3 levels correlated with specific VWT, highlighting their potential utility in identifying patients at greater risk of venous inflammation and thrombosis. P-selectin has been identified as an important factor in inflammation and thrombosis due to its established role in leucocyte and platelet adhesion in numerous previous studies [20]. Growing evidence indicates the contribution of NETs formation (NETosis), driven by protein-arginine deiminase type 4, to thrombosis, ischaemia and atherosclerosis [21]. A recent study has associated CH3 with an increased risk of thrombosis in cancer patients, independent of other factors [22]. These biomarkers not only differentiate SLE-APS patients from other groups but also offer a window into the complex interplay between inflammation, endothelial dysfunction and coagulation in SLE.

The finding that portal VWT was the only vascular measurement significantly correlated with all three biomarkers—P-selectin, GDF-15 and CH-3—highlights its potential as a unique marker of subclinical vascular pathology in SLE. While femoral artery and femoral vein thickness were also associated with selected biomarkers, the consistency and strength of correlations observed with the portal vein suggest a more generalized or systemic vascular involvement that may be captured in this region. P-selectin and GDF-15 have both been implicated in endothelial activation, inflammation and thrombo-inflammatory processes [23, 24], while CH-3 is emerging as a potential marker of vascular remodelling [25]. The simultaneous association of these markers with a single vascular site may reflect a cumulative burden of endothelial dysfunction, immune-mediated vascular injury and prothrombotic potential, particularly in venous beds that may be more vulnerable to inflammatory insults. Given that the portal vein is a central conduit draining splanchnic circulation, increased wall thickness in this region may also represent early signs of systemic vasculopathy, possibly preceding clinical thrombotic events. These observations warrant further prospective studies to evaluate whether portal vein measurements can serve as a surrogate marker for vascular risk stratification or treatment response monitoring in patients with SLE.

Interestingly, there were no significant differences in VWT or biomarker levels between clinically active and inactive SLE patients according to SLEDAI. In a previous study evaluating the risk of venous recurrence in SLE patients, no significant association was found between an increase in SLEDAI scores and the risk of venous thrombosis recurrence [26]. Also, another study showed no association between markers of endothelial cell activation and the presence of LA or a history of thrombosis in SLE patients [27]. Collectively, these findings suggest that subclinical venous involvement may occur independently of overall disease activity.

In this context, it is worth noting that LA testing in our study was analysed as a continuous variable, based on the raw numeric output of phospholipid-sensitive coagulation assays. Although this approach is not conventional in clinical practice, it allowed for the evaluation of potential dose-dependent effects or subclinical thresholds of procoagulant activity that may not be captured using binary classification. We acknowledge that further studies are warranted to assess the validity and reproducibility of this strategy in larger cohorts. Taken together, these results raise the possibility that vascular inflammation in SLE represents a distinct pathological process, potentially requiring targeted therapeutic strategies.

The SLE-APS group exhibited significantly higher SDI scores than the other SLE subgroups. SDI showed positive associations with multiple ultrasonographic measurements of VWT, particularly the thickness of the left jugular vein and the diameter of the portal vein. In contrast, no significant correlations were observed between SDI and circulating biomarkers such as P-selectin, GDF15 and CH3. These findings may reflect a link between cumulative organ damage and chronic structural venous changes, particularly in patients with longstanding or severe disease manifestations such as APS. However, the lack of association between SDI and circulating biomarkers suggests a more complex and possibly independent pathway between biochemical inflammation and irreversible vascular remodelling.

Despite the promising findings, certain limitations should be addressed. The cross-sectional design precludes establishing causality between elevated biomarker levels and venous wall thickening. Besides, the potential effect of the patient’s immunosuppressive treatment on VWT has not been investigated. Longitudinal studies are necessary to determine whether changes in these markers correlate with disease progression or predict thrombotic events. Additionally, the relatively small sample size, particularly within subgroups, limits the generalizability of the results.

Future research should aim to validate the utility of P-selectin, GDF15 and CH3 as biomarkers in larger, more diverse SLE cohorts. Moreover, exploring their interactions with other inflammatory mediators and vascular processes could yield deeper insights into the pathogenesis of venous complications in SLE. Therapeutic strategies targeting these biomarkers, such as agents modulating P-selectin activity or GDF15 expression, may offer novel approaches for mitigating vascular inflammation and thrombotic risk in SLE patients.

This study demonstrates that VWT serves as a marker of subclinical venous inflammation in SLE patients. In particular, the elevated levels of P-selectin, GDF-15 and CH3 in patients with APS suggest that these biomarkers may be useful in assessing thrombotic risk.

As a conclusion, we identified increased VWT in patients with SLE, which may indicate subclinical venous involvement—a previously underrecognized vascular feature in SLE. Associations between VWT and thrombosis, as well as elevated levels of P-selectin, GDF15 and CH3, support a link between vascular structural changes and prothrombotic states. However, in the absence of direct histopathologic or functional imaging data, it remains uncertain whether VWT reflects active inflammation, chronic vascular remodelling or cumulative damage. Notably, these findings were consistent across disease activity levels, suggesting that such vascular changes may occur independently of acute disease flares. Our results underscore the potential utility of integrating biochemical and imaging markers to better characterize vascular risk in SLE, but further longitudinal and mechanistic studies are warranted.

## Supplementary Material

keaf468_Supplementary_Data

## Data Availability

The data that support the findings of this study are available from the corresponding author upon reasonable request.
