# Supplementary material for: A new characteristic of SLE: subclinical vein involvement–an in-depth biochemical and imaging study
Source: Rheumatology (Oxford). 2025 Sep 5;65(1):keaf468. doi: 10.1093/rheumatology/keaf468 (PMC12862360; doi:10.1093/rheumatology/keaf468)

|  | **SLE- APS**  **(n=20)** | **SLE w/ (+) APA (n=18)** | **SLE w/o APA & APS positivity(n=30)** | **p** |
| --- | --- | --- | --- | --- |
| **Neurologic involvement(n(%))**  **Headache**  **Optic neuritis**  **Sinus thrombosis**  **Epilepsy**  **CVA** | 10(50)  -  3  3  1  5 | 3(16)  1  -  -  1  1 | 3(10)  -  -  -  1  2 | **0.008^1^** |
| **Arthritis** | 18(90) | 15(83) | 28 (93) | 0.5 |
| **Pulmonary involvement (n(%))** | 6(30) | 2(11) | 10(33) | 0.2 |
| **Renal involvement (n(%))*** | 7(35) | 5(27) | 14(46) | 0.4 |
| **GI involvement (n(%))** | 3(15) | - | 1(3.3) | NA |
| **Ocular involvement (n(%))** | 1(5) | 1(5.5) | 2(6.6) | NA |
| **Hematologic involvement (n(%))** | 11(55) | 7(38) | 8(26.6) | 0.1 |

**Supplementary Table S1.** Comparative Analysis of Clinical Manifestations of SLE Across Study Subgroups

^1^Difference is caused by patients with clinical APS group

APS: Antiphospholipid syndrome, APA: Antiphospholipid antibody, CVA: Cerebrovascular accident, PAP: Pulmonary arterial pressure, SLE: Systemic Lupus Erythematosus, CVA: Cerebrovascular accident, GI: Gastrointestinal

**Supplementary Table S2.** Comparison of vessel wall thicknesses between SLE patients when analyzed about clinical activity

|  | **Patients at the active period (n=26)** | **Patients at remission (n=42)** | **p** |
| --- | --- | --- | --- |
| **Common femoral vein (cm, median(IQR))** |  |  |  |
| **Right** | 0.4(0.2) | 0.4(0.18) | 0.6 |
| **Left** | 0.4(0.18) | 0.4(0.3) | 0.3 |
| **Superficial femoral vein (cm, median(IQR))** |  |  |  |
| **Right** | 0.4(0.18) | 0.4(0.2) | 0.4 |
| **Left** | 0.3(0.2) | 0.4(0.2) | 0.2 |
| **Portal vein (cm, median(IQR))** | 1.2(0.3) | 1.25(0.2) | 0.6 |
| **Juguler vein (cm, median(IQR))** |  |  |  |
| **Right** | 0.3(0.18) | 0.3(0) | 0.1 |
| **Left** | 0.3(0.18) | 0.3(0) | 0.5 |
| **Femoral artery (cm, median(IQR))** |  |  |  |
| **Right** | 0.6(0.1) | 0.6(0.17) | 0.6 |
| **Left** | 0.6(0.2) | 0.6(0.1) | 0.3 |
| **D-dime**r**(µg/Ml,median (IQR))** | 0.4(0.3) | 0.5(0.2) | 0.7 |
| **P-Selectin(ng/mL, median(IQR))** | 72(27) | 59(43) | 0.6 |
| **HDGF15(ng/mL, median(IQR))** | 1325(422) | 1211(438) | 0.9 |
| **CH3(ng/mL, median(IQR))** | 200(83) | 216(96) | 0.4 |

SLE: Systemic lupus erythematosus, HDGF: Human derived growth factor, CH: Citrillunated histon, IQR: Interquartile range

## **Supplementary Table S3.** Comparison of Vein Wall Thickness Between Total SLE Cohort and Controls

| **Vein Region** | **SLE Median (IQR), n=68*** | **Control Median (IQR), n=42**** | **p-value** |
| --- | --- | --- | --- |
| Right CFV | 0.50 (0.40–0.60) | 0.30 (0.30–0.40) | **< 0.001** |
| Left CFV | 0.50 (0.40–0.60) | 0.30 (0.30–0.40) | **< 0.001** |
| Right SFV | 0.50 (0.40–0.60) | 0.30 (0.30–0.40) | **< 0.001** |
| Left SFV | 0.50 (0.40–0.60) | 0.30 (0.30–0.40) | **< 0.001** |
| Portal Vein | 1.10 (1.00–1.30) | 0.90 (0.80–1.00) | **0.002** |
| Right JV | 0.30 (0.20–0.40) | 0.20 (0.20–0.30) | **< 0.001** |
| Left JV | 0.30 (0.20–0.40) | 0.20 (0.20–0.30) | **< 0.001** |

*SLE group refers to all SLE patients included in the study not regarding antiphospholipid antibody or antiphospholipid antibody syndrome status

**Control group refers to the sum of participant in both healthy controls and rheumatoid arthritis groups.
SLE = Systemic Lupus Erythematosus; RA = Rheumatoid Arthritis; CFV = Common Femoral Vein; SFV = Superficial Femoral Vein; JV = Jugular Vein. p-values are from two-sided Mann–Whitney U tests.

**Supplementary Table S4.** Comparison of vascular wall thickness and biomarker levels according to treatment exposure

| **Treatment (patients (n))** | **Parameter** | **p-value** |
| --- | --- | --- |
| **MPZ use (max dose is 6 mg/day)**  **n = 30** | Portalvein | 0.58 |
|  | RightCFV | 0.87 |
|  | LeftCFV | 0.83 |
|  | RightSFV | 0.71 |
|  | LeftSFV | 0.26 |
|  | RightJV | 0.55 |
|  | LeftJV | 0.91 |
|  | p_selectin | 0.38 |
|  | HGDF15 | 0.36 |
|  | CH3 | 0.6 |
| **MMF use (2000 mg/day)**  **(n = 15)** | Portalvein | 0.15 |
|  | RightCFV | 0.49 |
|  | LeftCFV | 0.78 |
|  | RightSFV | 0.17 |
|  | LeftSFV | 0.24 |
|  | RightJV | 0.49 |
|  | LeftJV | 0.49 |
|  | p_selectin | 0.31 |
|  | HGDF15 | 0.11 |
|  | CH3 | 0.46 |
| **Rituximab use (1000 mg/6 months)**  **(n = 7)** | Portalvein | 0.36 |
|  | RightCFV | 0.36 |
|  | LeftCFV | 0.23 |
|  | RightSFV | 0.4 |
|  | LeftSFV | 0.1 |
|  | RightJV | 0.5 |
|  | LeftJV | 0.4 |
|  | p_selectin | 0.5 |
|  | HGDF15 | 0.1 |
|  | CH3 | 0.2 |
| **HCQ use (200-400 mg/day)**  **n = 54** | Portalvein | 0.8 |
|  | RightCFV | 0.3 |
|  | LeftCFV | 0.3 |
|  | RightSFV | 0.3 |
|  | LeftSFV | 0.5 |
|  | RightJV | 0.5 |
|  | LeftJV | 0.5 |
|  | p_selectin | 0.7 |
|  | HGDF15 | 0.1 |
|  | CH3 | 0.2 |
| **AZA use (200 mg/day)**  **n = 10** | Portalvein | 0.7 |
|  | RightCFV | 0.7 |
|  | LeftCFV | 0.5 |
|  | RightSFV | 0.6 |
|  | LeftSFV | 0.3 |
|  | RightJV | 0.9 |
|  | LeftJV | 0.8 |
|  | p_selectin | 0.8 |
|  | HGDF15 | 0.6 |
|  | CH3 | 0.1 |
| **MTX use (max dose of 20 mg/week)**  **n = 6** | Portalvein | 0.9 |
|  | RightCFV | 0.1 |
|  | LeftCFV | 0.09 |
|  | RightSFV | 0.9 |
|  | LeftSFV | 0.7 |
|  | RightJV | 0.6 |
|  | LeftJV | 0.1 |
|  | p_selectin | 0.3 |
|  | HGDF15 | 0.4 |
|  | CH3 | 0.1 |
| **CNI use (all was cyclosporin, max dose of 150 mg/day)**  **n = 8** | Portalvein | 0.6 |
|  | RightCFV | 0.9 |
|  | LeftCFV | 0.5 |
|  | RightSFV | 1.0 |
|  | LeftSFV | 0.8 |
|  | RightJV | 0.9 |
|  | LeftJV | 0.9 |
|  | p_selectin | 0.3 |
|  | HGDF15 | 0.6 |
|  | CH3 | 0.7 |

Note: No statistically significant differences were found between treated and untreated groups in any of the evaluated parameters.
Abbreviations: MMF, mycophenolate mofetil; AZA, azathioprine; CNI, calcineurine inhibitors; MTX, methotrexate; HCQ, hydroxychloroquine, CFV: common femoral vein, SFV: superficial femoral vein, JV: jugular vein, HDGF-15: human growth differentiation factor, CH: citrillunated histone

**Supplementary Table S5.** Correlation analyses of vessel wall thicknesses

|  |  | Right CFV | Left CFV | Right SFV | Left SFV | Portal Vein | Right JV | Left JV | Right FA | Left FA |
| --- | --- | --- | --- | --- | --- | --- | --- | --- | --- | --- |
| Right CFV | r  p- | 1 |  |  |  |  |  |  |  |  |
| Left CFV | R  P | 0.83  **<0.001** | 1 |  |  |  |  |  |  |  |
| Right SFV | R  p | 0.84  **<0.001** | 0.87  **<0.001** | 1 |  |  |  |  |  |  |
| Left SFV | R  p | 0.83  **<0.001** | 0.86  **<0.001** | 0.9  **<0.001** | 1 |  |  |  |  |  |
| Portal Vein | R  p | 0.64  **<0.001** | 0.65  **<0.001** | 0.7  **<0.001** | 0.65  **<0.001** | 1 |  |  |  |  |
| Right JV | R  p | 0.72  **<0.001** | 0.74  **<0.001** | 0.74  **<0.001** | 0.74  **<0.001** | 0.66  **<0.001** | 1 |  |  |  |
| Left JV | R  p | 0.76  **<0.001** | 0.77  **<0.001** | 0.77  **<0.001** | 0.76  **<0.001** | 0.73  **<0.001** | 0.89  **<0.001** | 1 |  |  |
| Right FA | R  p | 0.57  **<0.001** | 0.56  **<0.001** | 0.57  **<0.001** | 0.58  **<0.001** | 0.47  **<0.001** | 0.5  **<0.001** | 0.52  **<0.001** | 1 |  |
| Left FA | R  p | 0.55  **<0.001** | 0.54  **<0.001** | 0.6  **<0.001** | 0.58  **<0.001** | 0.48  **<0.001** | 0.46  **<0.001** | 0.43  **<0.001** | 0.73  **<0.001** | 1 |

CFV: Common Femoral Vein, SFV: Superficial Femoral Vein, JV: Juguler Vein, FA: Femoral Artery

**Supplementary Table S6.**  Correlation analyses of vessel wall thicknesses with P-selectin, HDGF15, CH3, and D-dimer Levels (n= 68).

|  |  | GDF-15 | P-Selectin | CH3 | D-dimer |
| --- | --- | --- | --- | --- | --- |
| Right CFV | r  p | 0.12  0.24 | 0.76  0.4 | 0.12  0.26 | 0.11  0.9 |
| Left CFV | r  P | **0.2**  **0.05** | 0.15  0.17 | 0.21  0.056 | 0.03  0.7 |
| Right SFV | R  p | **0.21**  **0.04** | 0.14  0.1 | 0.19  0.08 | 0.09  0.3 |
| Left SFV | R  p | 0.18  0.1 | 0.07  0.5 | 0.15  0.16 | 0.05  0.63 |
| Portal Vein | R  p | **0.27**  **0.01** | **0.26**  **0.02** | **0.23**  **0.04** | 0.12  0.3 |
| Right JV | R  p | **0.18**  **0.09** | 0.04  0.7 | 0.14  0.18 | 0.06  0.9 |
| Left JV | R  p | **0.23**  **0.03** | 0.1  0.3 | 0.19  0.06 | 0.21  0.8 |
| Right FA | R  p | 0.06  0.5 | 0.03  0.7 | 0.03  0.7 | 0.05  0.6 |
| Left FA | R  p | 0.06  0.59 | 0.009  0.9 | 0.03  0.7 | 0.14  0.2 |

CFV: Common Femoral Vein, SFV: Superficial Femoral Vein, JV: Jugular Vein, FA: Femoral Artery, HDGF: Human Derived Growth Factor, CH: Citrullinated Histone

**Supplementary Table S7.** Correlation analyses of HDGF15, CH3, and P-selectin levels with other parameters studied in serum samples (n:108)

|  |  | HDGF15 | CH3 | P-selectin | D-dimer |
| --- | --- | --- | --- | --- | --- |
| CH3 | r  p | **0.85**  **<0.001** | 1 |  |  |
| P-selectin | r  p | **0.81**  **<0.001** | **0.84**  **<0.001** | 1 |  |
| D-dimer | R  p | 0.1  0.3 | -0.12  0.27 | -0.06  0.5 | 1 |
| Antibeta_2_ GPI IgM | R  p | 0.2  0.06 | **0.28**  **0.009** | 0.2  0.06 | 0.1  0.07 |
| Antibeta_2_ GPI IgG | R  p | **0.28**  **0.01** | **0.3**  **0.004** | **0.35**  **0.001** | 0.02  0.8 |
| ACA IgM | R  p | **0.26**  **0.01** | **0.2**  **0.02** | **0.2**  **0.03** | 0.1  0.8 |
| ACA IgG | R  p | **0.3**  **0.005** | **0.2**  **0.03** | **0.3**  **0.006** | 0.2  0.07 |
| LA | R  p | 0.2  0.07 | 0.1  0.08 | 0.1  0.08 | 0.03  0.7 |

CFV: Common Femoral Vein, SFV: Superficial Femoral Vein, JV: Jugular Vein, FA: Femoral Artery, ACA: Anticardiolipin, LA: Lupus Anticoagulant, HDGF: Human Derived Growth Factor, CH: Citrillunated Histon, GPI: Glycoprotein I

**Supplementary Figure S1.** Correlation of [S1] age with vein and artery wall thickness and [S2] BMI with P-selection and GDF15 levels.


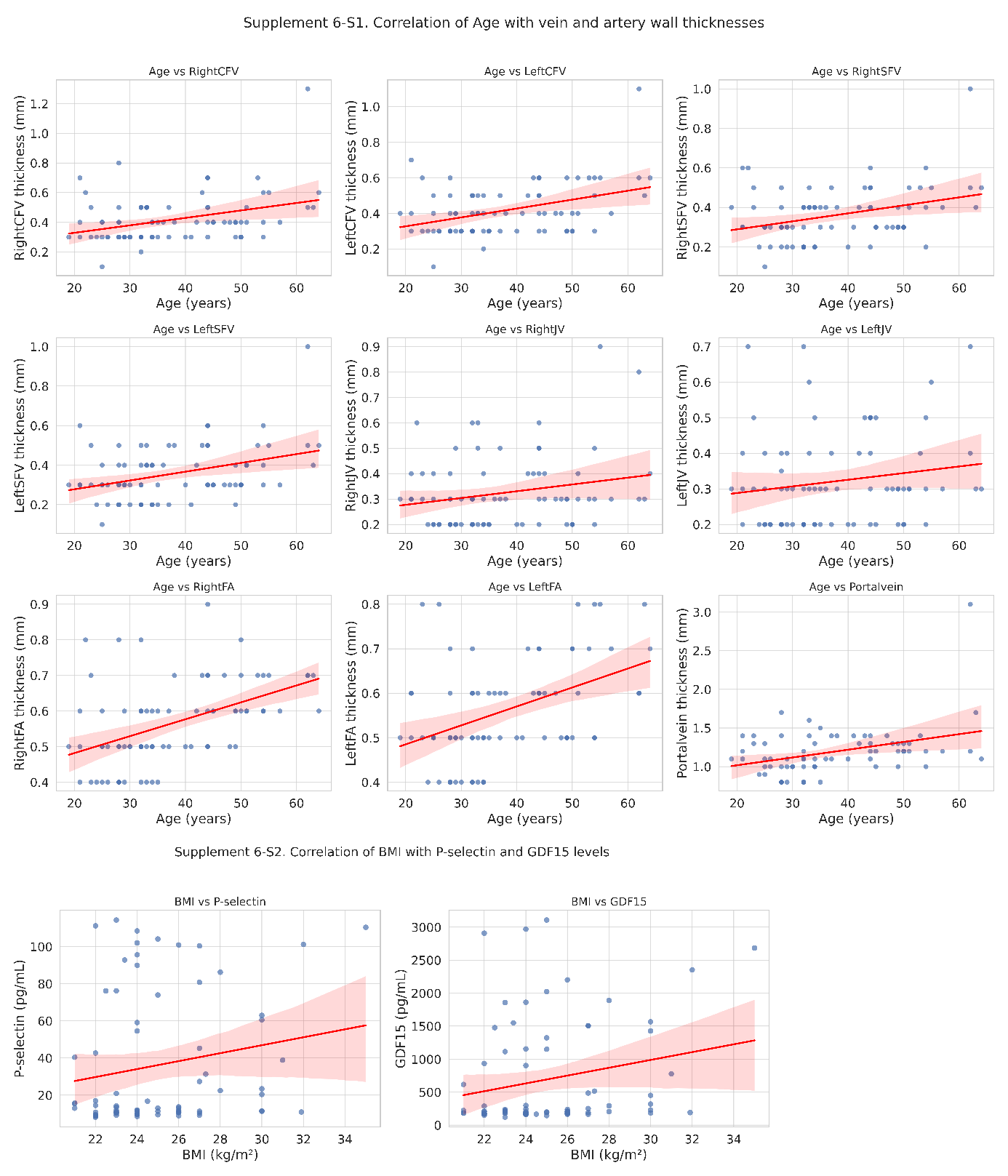

Supplement: keaf468_Supplementary_Data [file keaf468_supplementary_data.docx]
